# Silent Saboteurs: Decoding Mycotoxins—From Chemistry and Prevalence to Health Risks, Detection, Management and Emerging Frontiers

**DOI:** 10.3390/jof11120840

**Published:** 2025-11-27

**Authors:** Kasun M. Thambugala, Dilakshini Dayananda, Samawansha Tennakoon, Hiruni Harischandra, Pamoda Jayatunga, Nissanka de Silva, Asanthi Dhanusha, Sahan Madusanka, Dinushani A. Daranagama, Madhusha Gonapaladeniya, Sukanya Haituk, Ratchadawan Cheewangkoon

**Affiliations:** 1 Genetics and Molecular Biology Unit, Faculty of Applied Sciences, University of Sri Jayewardenepura, Nugegoda 10250, Sri Lanka; dilakshini@sci.sjp.ac.lk (D.D.); samawansha@sci.sjp.ac.lk (S.T.); hirunih@sci.sjp.ac.lk (H.H.); pamoda@sci.sjp.ac.lk (P.J.); nissanka@sci.sjp.ac.lk (N.d.S.); asanthidhanusha@gmail.com (A.D.); madusankarex99@gmail.com (S.M.); 2 Center for Plant Materials and Herbal Products Research, University of Sri Jayewardenepura, Nugegoda 10250, Sri Lanka; 3 Center for Biotechnology, Department of Zoology, University of Sri Jayewardenepura, Nugegoda 10250, Sri Lanka; 4 Department of Plant and Molecular Biology, Faculty of Science, University of Kelaniya, Kelaniya 11300, Sri Lanka; anupamad@kln.ac.lk; 5 Department of Medical Laboratory Sciences, Faculty of Allied Health Sciences, University of Sri Jayewardenepura, Nugegoda 10250, Sri Lanka; gonapaladeniya@sjp.ac.lk; 6 Office of the Research Administration, Chiang Mai University, Chiang Mai 50200, Thailand; sukanya.h@cmu.ac.th; 7 Department of Entomology and Plant Pathology, Faculty of Agriculture, Chiang Mai University, Chiang Mai 50200, Thailand

**Keywords:** mycotoxins, biosynthesis pathways, food safety, fungal metabolites, machine learning, risk assessment

## Abstract

Mycotoxins, toxic secondary metabolites produced by filamentous fungi, pose significant threats to global food safety, public health, and agricultural sustainability. This review summarizes the classification, biosynthesis, chemistry, and mechanisms of action of these compounds, and highlights their global prevalence and the serious health consequences of both acute and chronic exposure. Despite decades of research, substantial gaps remain in effective surveillance, prevention, and risk management. Traditional control and detection strategies, although valuable, are often limited by their sensitivity, high costs, and inadequate field applicability. Addressing these gaps, this review emphasizes the potential of emerging technologies, particularly the integration of artificial intelligence (AI) and machine learning (ML) with advanced sensing platforms, to revolutionize mycotoxin detection. These innovations offer enhanced precision, real-time monitoring, and predictive modelling capabilities, paving the way for proactive food safety systems. By critically evaluating current knowledge and exploring future-oriented solutions, this review highlights the urgent need for interdisciplinary approaches that integrate molecular insights, biotechnological advancements, and digital technologies. Finally, we emphasize that adopting these novel strategies is essential to overcoming the silent yet profound global impact of mycotoxins.

## 1. Introduction

Mycotoxins are a chemically heterogeneous class of toxic secondary metabolites generated by a wide range of filamentous fungi [1,2], including *Alternaria*, *Aspergillus*, *Fusarium*, and *Penicillium* species. These compounds are generally characterized by low molecular weights below 1000 Da [3]. More than 500 mycotoxins have been identified to date [4]; however, six major groups; aflatoxins, fumonisins, ochratoxins, trichothecenes, zearalenone, and patulin, are most prevalent and represent the greatest threat to food safety [5]. Mycotoxins are not essential for fungal growth but confer ecological advantages, such as inhibiting the competing microorganisms and facilitating substrate colonization.

**Figure 1:**
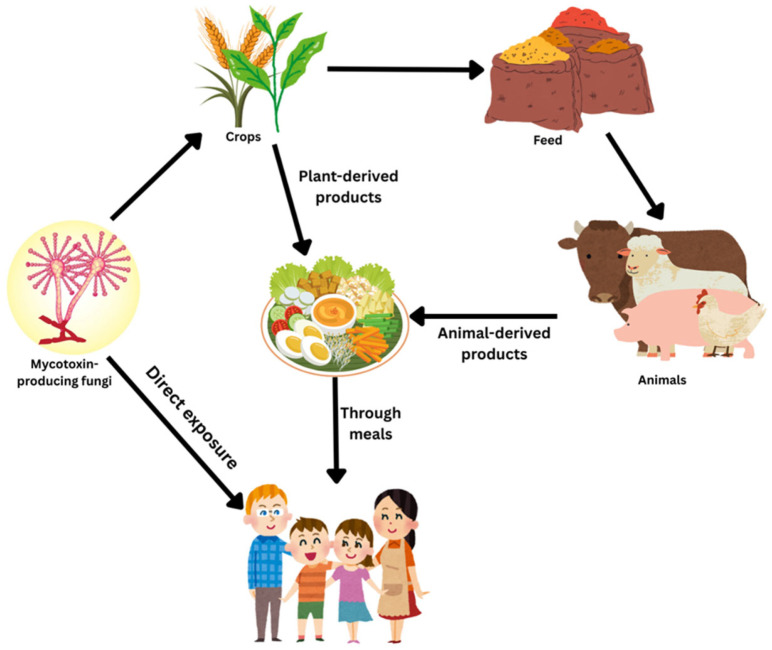
Mycotoxins contamination pathways.

Mycotoxins can contaminate a wide range of agricultural commodities, including cereals, legumes, nuts, and spices. This contamination of crops can occur during pre-harvest, post-harvest, and storage (Figure 1). Most mycotoxins are known to be heat stable and therefore are not destroyed during normal food processing and cooking procedures. They require prolonged exposure to temperatures above 120 °C to be destroyed, with some, such as ochratoxins, surviving temperatures as high as 250 °C. Moreover, multi-mycotoxin contamination with binary, ternary and quaternary combinations is increasing worldwide [5,6,7], leading to heightened health risks due to synergistic toxic effects, greater challenges in food safety management, and increased economic losses in agriculture [4,8].

Mycotoxins are toxic to vertebrates and other animal groups even at low concentrations. Exposure to mycotoxins through ingestion, skin or eye contact, or inhalation [9] can lead to mycotoxicosis. The best-known mycotoxicosis cases demonstrate acute effects leading to the deterioration of the liver and kidneys of humans and animals, sometimes leading to death within a short period following the exposure [10,11,12]. However, chronic exposure to mycotoxins can result in cancer development, neurological disorders, and immune suppression [10,13,14,15]. Aflatoxins, ochratoxins, patulin, fumonisins, zearalenones, and trichothecenes produced by *Aspergillus*, *Fusarium*, and *Penicillium* species have been the focus of studies due to their impact on human and animal health [13,14,16]. These compounds are mainly found in cereal grains such as maize, wheat and barley. Aflatoxins and ochratoxins are the most common mycotoxins found in bread, while ochratoxins are the most frequently found mycotoxin in wine [17,18]. Fumonisins are mainly found in maize and maize-derived products, while patulins are mainly found in apples and apple products such as apple juice and cider [13,19,20]. These aflatoxins and ochratoxins are known for their genotoxic and carcinogenic effects [13], while patulin and zearalenones are known to exert only genotoxicity and fumonisins only carcinogenicity. Aflatoxins, ochratoxins, fumonisins, and patulin cause both nephropathy and hepatotoxicity [21,22]. All six mycotoxin classes can cross the blood–brain barrier, potentially affecting the functions of the brain, while all except fumonisins can cross the placenta, thereby potentially affecting fetal development [23,24,25]. Ochratoxins, Zearalenones and trichothecenes are known mainly for causing reproductive disorders [26,27,28].

Besides these negative impacts on human and animal health, mycotoxins also cause a significant economic burden due to the loss of crops such as maize, corn, and peanuts, as well as livestock [13,29]. Studies estimate an annual economic burden of around $1 billion in some countries, and project that it could exceed $500 billion if the current guidelines for fumonisin and aflatoxin, approved by the United States Food and Drug Administration (FDA), are adopted worldwide [30].

Emerging technologies, including genomics, metabolomics, and artificial intelligence-based predictive models, are being increasingly employed to understand and monitor mycotoxin contamination, providing opportunities for improved food safety and public health protection. Hence, a comprehensive knowledge of mycotoxin types, biosynthetic mechanisms, and modes of action is critical for developing effective detection methods, risk assessment strategies, and mitigation approaches in food and feed systems. Prevention and removal of mycotoxin contaminations require careful monitoring, adherence to good agricultural practices, effective post-harvest management, and detoxification. It is imperative to employ integrated approaches to prevent and control mycotoxin contamination to safeguard human and animal health, food security, and economic stability.

This review provides a comprehensive overview of the current knowledge on mycotoxins, encompassing their classification, chemical structures, biosynthetic pathways, and mechanisms of toxicity. It also highlights global prevalence patterns, associated health risks, and the limitations of traditional detection and management strategies. Furthermore, the review emphasizes emerging technologies, such as artificial intelligence, machine learning, advanced sensing platforms, and omics-based approaches, as innovative tools for enhanced detection, risk assessment, and mitigation. By integrating insights from molecular biology, biotechnology, and digital technologies, this review aims to identify future research directions and emerging frontiers that can strengthen proactive mycotoxin management, improve food safety, and reduce the global health and economic burden posed by these silent yet pervasive threats.

## 2. Mycotoxin Types, Chemistry, Biosynthesis, and Mechanism of Action

Structurally, mycotoxins encompass six (06) main types, including aflatoxins, ochratoxins, trichothecenes, fumonisins, zearalenone, and patulin, each with distinct physicochemical properties and toxicological profiles. These main mycotoxin groups contain functional moieties such as lactones, epoxide groups, ketone and hydroxyl substituents, and aromatic rings, which underpin their biological activity and toxic potential [31]. Different mycotoxins exhibit a spectrum of polarity from lipophilic to moderately polar, which affects their bioavailability and patterns of tissue accumulation [32].

The biosynthesis of mycotoxins involves complex enzymatic pathways often regulated by environmental factors, such as temperature, humidity, substrate composition, and oxidative stress. Polyketide synthases, non-ribosomal peptide synthetases, and terpenoid synthases are among the key enzymes orchestrating their assembly, frequently organized within gene clusters whose expression is tightly regulated at transcriptional and post-transcriptional levels. Advances in molecular biology have revealed regulatory networks, including transcription factors and epigenetic modifications, that control mycotoxin production in response to biotic and abiotic signals.

Mechanistically, mycotoxins exert toxicity through diverse cellular targets and pathways. For instance, aflatoxins intercalate with DNA and induce mutagenic adducts, whereas ochratoxins inhibit protein synthesis and promote oxidative stress. Further, trichothecenes block ribosomal function, impairing translation. Fumonisins disrupt sphingolipid metabolism, leading to cellular apoptosis, and zearalenone acts as an estrogenic mimic, interfering with reproductive physiology [33,34]. The interplay between the chemical structure of each mycotoxin and its molecular target underlies the variation in both acute and chronic toxic effects observed across different species.

The following section provides an overview of their chemistry, biosynthesis and mechanisms of action of the main types of mycotoxins.

### 2.1. Aflatoxins

Aflatoxins (AFs) are a group of mycotoxins primarily produced by *Aspergillus* species. Aflatoxins are the most significant mycotoxins in terms of their occurrence, human impact, toxicity, and abundance. *Aspergillus* species such as *A*. *flavus*, *A*. *bombycis*, *A*. *seudotamarii*, *A*. *nomius*, and *A*. *parasiticus*, can infest several crops, foods, and agricultural products, leading to the production of aflatoxins, which are classified as AFB_1_, AFB_2_, AFG_1_, AFG_2_, AFM_1_, and AFM_2_. The commonly known naturally occurring AF types, AFB1, AFB2, AFG1 and AFG2, are primarily produced by *A*. *flavus*, *A*. *parasiticus*, and *A*. *nomius* [35,36,37,38]. Aflatoxins commonly contaminate staple crops, including maize (corn), groundnuts (peanuts), tree nuts (pistachios, almonds, and walnuts), cottonseed, spices, dried fruits and rice, where the opportunistic pathogen, *A*. *flavus*, commonly thrives [22,39].

The biosynthesis of AFs involves a cascade of enzymatic and biochemical conversions, initiated from acetyl-CoA and malonyl-CoA, where dehydrogenase and NOR (Nitric Oxide Reductase) enzymes facilitate the reduction process [40,41]. Acetyl-CoA and malonyl-CoA are converted to hexanoyl-CoA, norsolorinic acid, averantin, 5”-hydroxyaverantin, averufin, versiconal, versicolorin A, demethylsterigmstocystin, streigmatocystin (SGT), O-methylsterigmatocystin, and dihydro-o-methylsterigmatocystin to produce type B and G AFs.

Of the different AF types, AFB1 is considered the most carcinogenic and genotoxic. Several studies demonstrate a higher cancer incidence rate due to the ingestion of dietary AFB1, suggesting it as a cancer biomarker. Furthermore, studies indicate a synergistic effect of AFB1 by microbial pathogens such as Hepatitis B Virus (HBV) and Hepatitis C Virus (HCV) in enhancing tumour initiation [37].

Metabolic activation of AFB1 occurs in the liver through conversion into a mutagenic and carcinogenic intermediate metabolite, AFB1-8,9-epoxide (AFBO) by cytochrome P450 (CYP450) microsomal enzymes [41]. In addition to the production of AFBO, cytochrome P450 metabolizes AFB1 into hydroxylation products such as aflatoxin H1 (AFH1), aflatoxin P1 (AFP1), aflatoxin M1 AFM1, aflatoxin Q1 (AFQ1), and aflatoxicol (AFL) [37]. Several in vivo studies confirm that AFM1 is the most carcinogenic of the hydrolysis products of AFB1 due to its ability to intercalate into DNA [37,42]. As the sole AFB1 metabolite is formed in the placenta, AFL is suggested to play a role in embryo development [37]. However, the exact roles of certain metabolic products of AFB1 are yet to be discovered.

**Figure 2:**
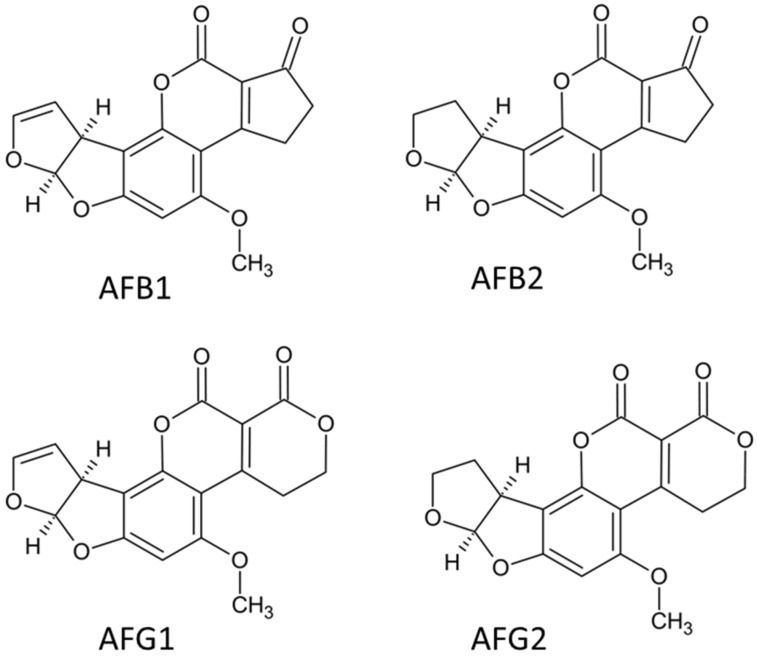
Structures of Aflatoxins (structure redrawn from PubChem SDF using ChemSketch (V 2024).

Aflatoxins share closely related structural frameworks representing a distinct group of highly oxygenated heterocyclic compounds [43] (Figure 2). Aflatoxins are classified as difuranocoumarin derivatives, structurally defined by a central coumarin core with a difuran substituent on one side and either a pentenyl ring or a six-membered lactone moiety on the other [44]. The four most prevalent aflatoxins—B1 (AFB1), B2 (AFB2), G1 (AFG1), and G2 (AFG2), differ primarily in the nature and position of specific functional groups within their molecular frameworks (Figure 2). The structure of AFB1 features a five-carbon cyclopentenone ring fusion to the coumarin lactone ring, which confers distinctive solubility characteristics in polar organic solvents and water [45]. The Group G aflatoxins are characterized by the presence of a six-membered delta-lactone ring instead of the cyclopentenone ring [46].

The pathway for biosynthesis of aflatoxins includes at least 23 enzymatic reactions and is controlled by a gene cluster of 70 kb. The cluster contains 25 co-regulated genes, with the essential regulatory gene aflR and its partner aflS [47]. Various intermediates such as norsolorinic acid, averantin, averufin, versiconal hemiacetal acetate, and versicolorin A are formed along the pathway, ultimately leading to the final aflatoxins [48]. Environmental conditions greatly affect aflatoxin production, with optimal conditions being temperatures of 25–30 °C, water activity greater than 0.95, and a pH of 6.0–8.0 [49].

Aflatoxins, especially AFB_1_, are highly potent natural carcinogens that mainly cause toxicity through DNA damage. Cytochrome P450 enzymes, particularly CYP1A2 and CYP3A4, convert AFB_1_ into AFB_1_-8,9-epoxide (AFBO). This reactive compound forms covalent bonds with guanine residues in DNA, leading to mutations [50]. Other affected targets include glutathione-S-transferases, mitochondrial respiratory complexes, and proteins involved in cell cycle checkpoints. Aflatoxins also induce oxidative stress, mitochondrial dysfunction [51] and inhibit protein synthesis. Detoxification processes include the hydrolysis of AFBO to AFB_1_-dihydrodiol or its reduction to aflatoxicol [52].

### 2.2. Ochratoxins

Ochratoxin A (OTA) is the most significant ochratoxin, mainly produced by *Aspergillus ochraceus*, *A*. *carbonarius*, and *Penicillium verrucosum*. Ochratoxins are known to contaminate cereals, coffee beans, grapes, dried fruits, wine, and spices. It is also known to persist through food processing and is often found in processed products such as bread, beer, and wine [22,53].

Ochratoxins are involved in causing nephropathy and cancer in humans, swine, and poultry [54]. Out of the several naturally occurring derivatives of ochratoxins, more widely spread OTA is the most hazardous type that can be found at all stages of the food chain [55]. Several studies confirm that OTA biosynthesis is regulated by several enzymes, including polyketide synthase (PKS), phenylalanine tRNA synthase, halogenase, methylase and ABC transporter gene [56,57].

It is a highly stable toxic fungal metabolite that can even withstand temperatures as high as 250 °C [58,59]. Inhibition of protein synthesis, impairment of the metabolic system, upregulation of membrane lipid peroxidation, disruption of calcium homeostasis, and DNA damage are some of the main mechanisms attributed to OTA regulation [54]. Although OTA has a high susceptibility to accumulate in kidneys, liver, skeletal muscles and brain, the rate of toxin absorption and its distribution to tissues vary depending on the species [60]. After injection of OTA, it binds to albumin with a high affinity (99.8%), and this binding determines the half-life of the plasma OTA as well as the toxicokinetics [55]. The kidney and liver are the most vulnerable towards OTA, where the cytochrome P450 plays a key role in forming (4S)-4-OH OTA and (4R)-4-OH OTA [60]. Although literature suggests multiorgan toxicity from OTA, there is a lack of definitive studies confirming its hepatotoxic role despite the liver’s crucial function in mycotoxin biotransformation and detoxification. However, in vitro studies suggest OTA-dependent apoptosis, reactive oxygen species (ROS) production, chromatin fragmentation and DNA damage in HepG2 liver cell line [61]. Further, in vivo studies demonstrate an OTA-dependent upregulation of inflammatory markers in duck livers and regulation of miRNA and protein expression in rats, offering insights into the roles of OTAs in nephrotoxicity and carcinogenesis [62,63,64].

**Figure 3:**
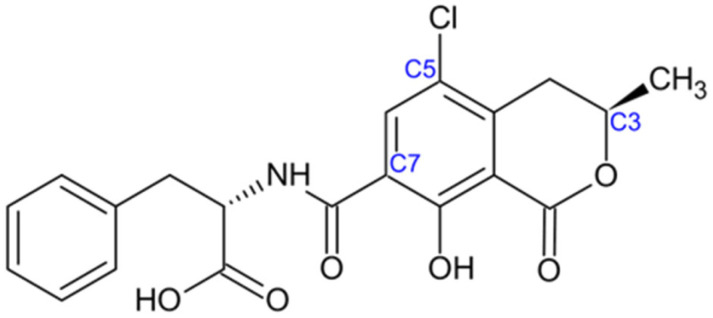
Structure of Ochratoxin A (structure redrawn from PubChem SDF using ChemSketch.

Ochratoxin A is a weak organic acid chemically identified as (2S)-2-[[(3R)-5-chloro-8-hydroxy-3-methyl-1-oxo-3,4-dihydroisochromene-7-carbonyl]amino]-3-phenylpropanoic acid. It is a phenylalanine-derived mycotoxin formed through the formal condensation of the amino group of L-phenylalanine with the carboxyl group of (3R)-5-chloro-8-hydroxy-3-methyl-1-oxo-3,4-dihydro-1H-2-benzopyran-7-carboxylic acid, commonly referred to as ochratoxin α (NCBI, 2025). Structurally, OTA consists of an isocoumarin-based polyketide framework conjugated to β-phenylalanine via a peptide bond (Figure 3). This unique structural arrangement, characterized by chlorination, hydroxylation, and a peptide linkage, underlies its chemical stability and contributes to its broad spectrum of biological activities [65].

The biosynthetic sequence combines L-phenylalanine with a polyketide chain derived from acetyl-CoA and malonyl-CoA. This process is supported by a polyketide synthase (PKS) and a non-ribosomal peptide synthetase (NRPS). The main 5 regulatory genes involved in this process are otaA, otaB, otaC, otaD and otaR1 [66]. The pathway ultimately produces dihydroisocoumarin, which combines with phenylalanine to create OTA.

Ochratoxin A has a long half-life of up to 35 days in humans. It enters cells through organic anion transporters, strongly binds to serum albumin (99%) and mainly affects the kidneys [67]. OTA inhibits protein synthesis by mimicking phenylalanine and competitively blocking phenylalanyl-tRNA synthetase [68]. Other harmful effects include genotoxicity, mitochondrial impairment, calcium imbalance, and negative effects on the immune system [69,70].

### 2.3. Fumonisins

Fumonisins pose a highly toxic threat towards human and animal health, easily affecting the host via the contaminated food, which could even lead to oesophageal cancer and neural tube defect [33]. Water-soluble, heat-stable secondary metabolite fumonisins are produced primarily by *Fusarium verticillioides* and *F*. *proliferatum*, and they can be categorized into four groups: Fumonisin A (FA), Fumonisin B (FB), Fumonisin C (FC), and Fumonisin P (FP), which further include 28 structural analogues [22,71]. Fumonisin B_1_ (FB_1_) is the most abundant and toxic fumonisin, which is known to contaminate maize and maize-based products in the world. Although the fumonisins show stability during food processing procedures, a significant toxin reduction has been observed by nixtamalization [33,72].

The toxicity of fumonisins towards an organism can be reflected via several processes, including impaired apoptosis, autophagy, immune toxicity, reproductive toxicity, tissue and organ toxicity, and carcinogenicity, which is suggested to be modulated through fumonisin-mediated sphingolipid metabolism [33,34]. Due to the structural similarities with sphingosine (diesters containing different polyols and glycerol tricarboxylic acids), fumonisins are classified under sphingosine-like mycotoxins [73]. Sphingolipids have been shown to regulate several cellular signalling events such as cell proliferation, differentiation, senescence, and apoptosis, in addition to being a key component of the biofilm, which is denoted by a layer of microorganisms bound and living extracellularly [33,74].

**Figure 4:**
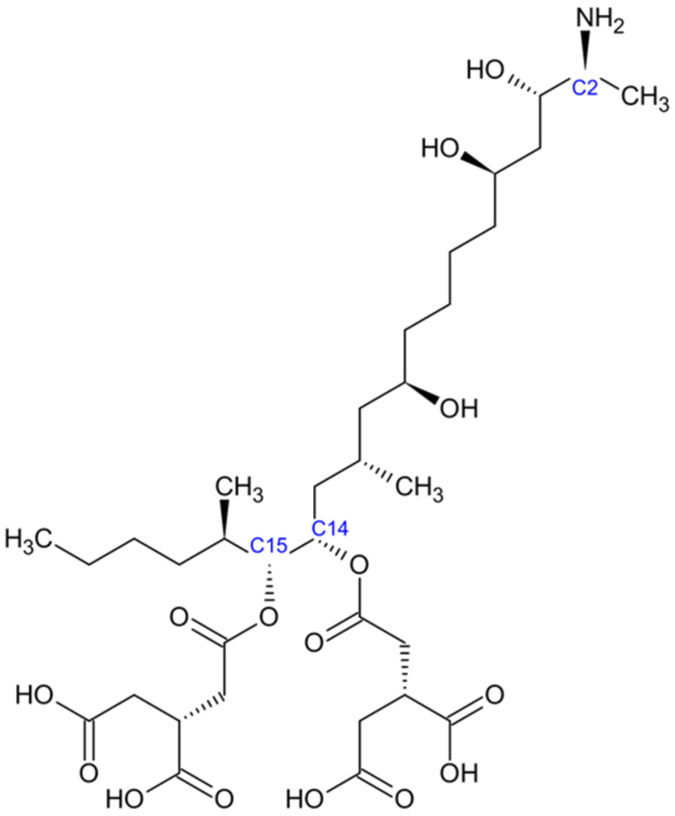
Structure of Fumonisin B1 (structure redrawn from PubChem SDF using ChemSketch.

Fumonisins exhibit structural features that are distinct from those of other mycotoxins. Their general architecture consists of a long 20-carbon chain bearing an amino (–NH_2_) group at the C-2 position and multiple hydroxyl (–OH) groups (Figure 4). The structure is also known as long-chain aminopolyols [33]. Notably, this structural arrangement resembles sphinganine and sphingosine, which are key constituents of cellular sphingolipids. This similarity explains their ability to disrupt sphingolipid metabolism [75]. Tricarballylic acid (TCA) side chains are esterified at specific positions along the carbon backbone, providing further structural definition to fumonisins. They are highly polar molecules, exhibiting good solubility in water and other polar solvents due to the presence of multiple reactive functional groups [76].

The biosynthesis pathway of Fumonisins involves combining alanine with a polyketide backbone from acetyl-CoA. This process is regulated by the FUM gene cluster, which has 15 genes. The FUM1 gene encodes a polyketide synthase that starts the condensation reaction [77]. The result is a long-chain amino polyol backbone that various enzymes further modify to form the complete fumonisin structure.

Fumonisins disrupt sphingolipid metabolism by blocking ceramide synthase [78]. This leads to the accumulation of sphinganine and sphingosine, which disrupt membrane integrity and signal transmission [79]. This disruption can cause apoptosis, oxidative injury, immune system problems, liver toxicity, pulmonary edema, and possible carcinogenic effects [80,81].

### 2.4. Trichothecenes

Trichothecenes constitute a large family of structurally related mycotoxins produced mainly by *Fusarium* species. Deoxynivalenol (DON, vomitoxin) is the most prevalent trichothecene, contaminating mostly wheat, barley, oats, and other cereals globally. T-2 and HT-2 types of trichothecenes are less common in crops but known to exhibit more toxic properties [22,82].

**Figure 5:**
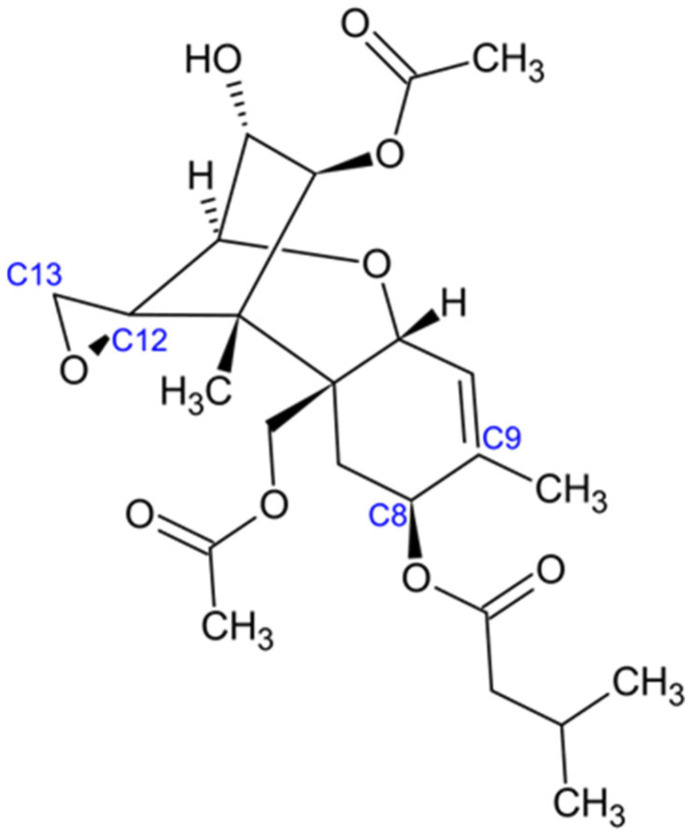
Structure of T-2 Toxin (structure redrawn from PubChem SDF using ChemSketch.

Trichothecenes constitute a diverse family of over 200 structurally related toxins characterized by a complex tetracyclic ring system. They are classified as sesquiterpenoids and share a conserved chemical framework known as 12,13-epoxytrichothec-9-ene (Figure 5, [83]).

The epoxide moiety at the C-12 and C-13 positions is critical for the toxic activity of trichothecenes. Structural diversity within this family arises from variations in substituents, such as hydroxyl or acetyl groups, attached at different positions of the core framework [84]. Based on these modifications, trichothecenes are subdivided into several types. Type A trichothecenes, including T-2 toxin and HT-2 toxin, lack a carbonyl group at C-8. Instead, both toxins carry an isovalerate substituent at this position [85]. In contrast, Type B trichothecenes, such as nivalenol, possess a carbonyl group at C-8, while Type C trichothecenes are distinguished by the presence of an additional epoxide ring at either C-7 or C-8. Thus, functional group substitutions play a central role in defining the chemical diversity and toxicological properties of the trichothecene family [85].

The biosynthesis pathway involves converting farnesyl pyrophosphate into trichodiene. This substrate undergoes several hydroxylation, acetylation, and other modifications via various enzymes, including trichodiene synthase, cytochrome P450 monooxygenases, and acetyltransferases. The production of trichothecenes is regulated by the TRI gene cluster [86,87]. Environmental factors and interactions with host plants greatly influence toxin production levels.

Trichothecenes are among the most potent natural inhibitors of protein synthesis in eukaryotes, binding to the 60S ribosomal subunit. This ribosome inhibition by trichothecenes triggers cellular reactions, including activating double-stranded RNA-activated protein kinase (PKR) and the p38 MAPK, JNK, and ERK signalling pathways. It also stimulates immediate early genes such as c-fos, c-jun, and c-myc [88]. A major outcome of trichothecenes is activating various apoptotic pathways, including intrinsic, extrinsic, and p53-dependent mechanisms [89].

Trichothecenes also have complex immunomodulatory effects, leading to increased pro-inflammatory responses and cytokine storms [87,90]. Additionally, trichothecenes are toxic to blood cell production [91], rapidly dividing intestinal cells [92], and the central nervous system [93].

### 2.5. Zearalenone

Zearalenone is mainly produced by *Fusarium graminearum* (also known as *Gibberella zeae*), *F*. *culmorum*, *F*. *cerealis*, *F*. *equiseti*, and *F*. *semitectum* [94]. These fungi commonly infect cereal crops such as maize, wheat, barley, oats, rice, and sorghum [95]. Zearalenone is also known for its estrogenic effects due to its structural similarity to estradiol [22,94].

**Figure 6:**
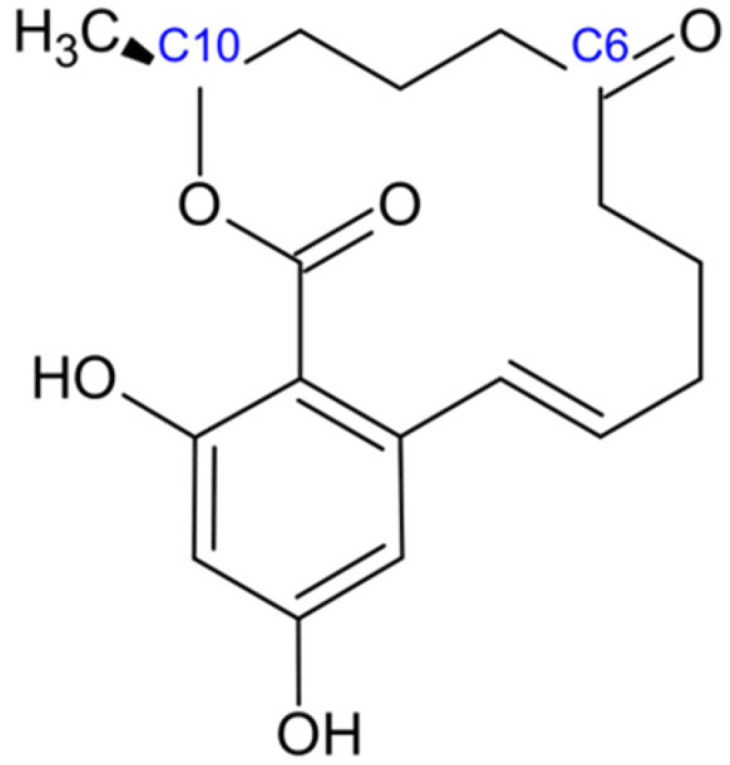
Structure of Zearalenone (structure redrawn from PubChem SDF using ChemSketch.

Zearalenone, a resorcylic acid lactone with the molecular formula C_18_H_22_O_5_ (Figure 6), is systematically designated as (4S,12E)-16,18-dihydroxy-4-methyl-3-oxabicyclo [12.4.0]octadeca-1(14),12,15,17-tetraene-2,8-dione under IUPAC nomenclature [95]. The core framework of zearalenone consists of a 14-membered lactone ring fused to a 1,3-dihydroxybenzene moiety. Within the macrocyclic lactone ring, a ketone group is present at the C-6 position, while a methyl group is located at the C-10 position [96]. The structural similarity between zearalenone and natural estrogens facilitates its ability to bind to mammalian estrogen receptors, thereby eliciting estrogenic effects [97]. Zearalenone is a weakly polar compound that is insoluble in water but readily dissolves in organic solutions [98]. It exhibits high thermostability and demonstrates resistance to degradation during common processing steps, including milling, extrusion, storage, and heating [97].

The biosynthetic pathway features polyketide synthesis and starts with acetyl-CoA and malonyl-CoA precursors. This process involves a series of enzymes, including polyketide synthases (PKS), with the ZEN1 gene cluster being crucial for core biosynthetic functions [99]. Factors like environmental stress, plant host characteristics, and the fungus’s genetics influence ZEN production levels in infected crops.

Zearalenone mainly acts as an endocrine disruptor by interacting with estrogen receptors α and β (ERα, ERβ), estrogen-related receptor γ (ERRγ), and cytochrome P450 enzymes (CYP1A1, CYP1A2), as well as 17β-hydroxysteroid dehydrogenase and aromatase (CYP19A1) [100].

When zearalenone binds to estrogen receptors, it activates estrogen response elements (EREs) in specific target genes. This activation can lead to reproductive and developmental toxicity, marked by reduced fertility, increased embryonic loss, and hyperplasia of mammary glands in females. In males, it results in decreased testosterone levels and lower sperm quality [94]. In vitro studies suggest that zearalenone may also have carcinogenic potential and could increase ROS levels [100,101]. Like other mycotoxins, zearalenone disrupts metabolic processes by altering steroid hormone ratios, affecting glucose and lipid metabolism, and modulating insulin sensitivity [102,103].

### 2.6. Patulin

Patulin is a mycotoxin classified as a polyketide. It is mainly produced by several species of *Penicillium*, *Aspergillus*, and *Byssochlamys*, including *P*. *expansum*, *A*. *clavatus*, *P*. *griseofulvum*, and *B*. *nivea* [104,105]. This mycotoxin is commonly found in damaged apples and apple-based products such as juice, cider, baby food, and other fruits, such as pears, apricots, and cherries [22,106,107].

**Figure 7:**
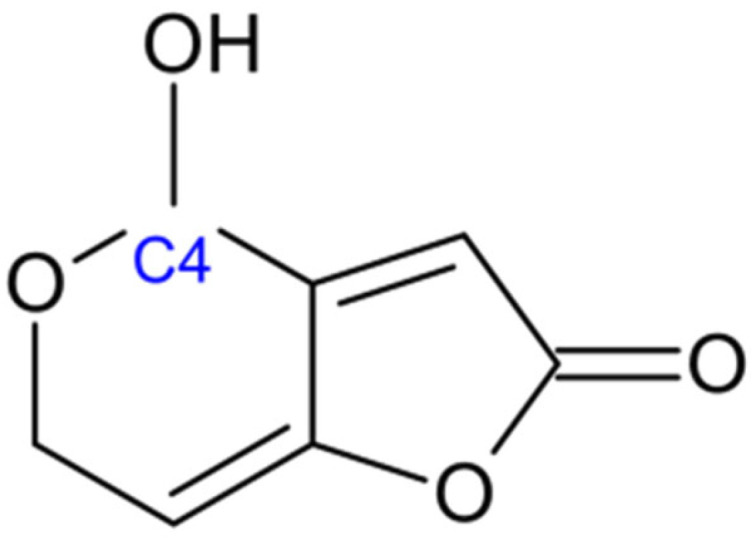
Structure of Patulin (structure redrawn from PubChem SDF using ChemSketch.

Patulin is a heterocyclic lactone with the molecular formula of C_7_H_6_O_4_. It is a relatively small mycotoxin, structurally characterized by a furan ring fused to a pyran ring [108]. The molecule contains a lactone functional group and two double bonds, as well as a hydroxyl group at the C-4 position of the pyran ring (Figure 7, [109]. This unsaturated framework, combined with the hydroxyl group, makes patulin highly reactive with biological molecules such as proteins and amino acids, which contributes to its toxicological effects [110].

The production of patulin follows a detailed polyketide synthesis pathway starting with glucose. This pathway involves several enzymatic steps, beginning with the formation of 6-methylsalicylic acid (6-MSA) by a polyketide synthase. Following that are oxidation reactions facilitated by various enzymes, including 6-MSA decarboxylase, isoepoxydon dehydrogenase, and patulin-forming enzymes [104,111]. Environmental conditions significantly impact patulin production, with ideal conditions being a pH of 3.5–5.5, temperatures between 20–25 °C, and high humidity [112].

Patulin causes toxicity by binding covalently to thiol (-SH) groups in cysteine residues, leading to enzyme inhibition and oxidative stress [113]. Key enzymes affected include glyceraldehyde-3-phosphate dehydrogenase, which disrupts glycolysis and glutathione metabolism. Furthermore, patulin also impairs mitochondrial activity by inhibiting enzymes in the Krebs cycle and components of the electron transport chain, reducing ATP production [96,114]. Its genotoxic effects include DNA adducts, chromosomal abnormalities, and micronuclei [115]. Although patulin is genotoxic, its potential carcinogenic effects on humans are still debated. Patulin also leads to lipid peroxidation, immune suppression, and disruption of cytokine activity [116,117].

## 3. Prevalence of Mycotoxins

Approximately 25% of the world’s crops are known to be contaminated with AFs, ochratoxins, fumonisins, and trichothecenes, being the most prevalent [22]. Furthermore, the co-occurrence of multiple mycotoxins in crops has also been reported [118]. Mycotoxin contamination exhibits distinct geographic patterns influenced by climate, agricultural practices, and storage conditions. Aflatoxins are predominantly found in tropical and subtropical regions of Africa, Asia, and Latin America [39]. Ochratoxins and trichothecenes are more prevalent in temperate regions of Europe and North America. Fumonisins are ubiquitous in maize-growing regions worldwide [119]. Agricultural commodities, including cereals, nuts, spices, and dried fruits, show the highest contamination frequencies. The ubiquitous nature of mycotoxin-producing fungi, combined with favourable environmental conditions during pre- and post-harvest periods, ensures their persistent presence in the global food supply chain, necessitating continuous monitoring and regulatory oversight [22,119].

The prevalence of mycotoxins in agricultural commodities varies widely depending on crop type, geographic location, climatic conditions, and agronomic practices. Legumes (Fabaceae) and maize (*Zea mays*) are among the most studied crops due to their dietary significance and susceptibility to fungal contamination. Studies across different regions have demonstrated that mycotoxin contamination in these crops is both widespread and complex, often involving multiple toxins and fungal species [113,120,121].

Aflatoxins production is closely linked to the growth of *Aspergillus flavus* and A. *parasiticus*, which thrive in warm, humid environments [39]. Sub-Saharan Africa reports the highest aflatoxin contamination rates globally, particularly in groundnut and maize samples under adverse climate conditions [122]. Ochratoxins and trichothecenes are more prevalent in temperate regions of Europe and North America [123]. Trichothecenes, including deoxynivalenol and T-2 toxin, show increased prevalence in wheat, barley, and oats grown in regions experiencing wet harvest conditions [124]. Fumonisins are abundant in maize-growing regions worldwide, with *Fusarium verticillioides* and *F*. *proliferatum* demonstrating remarkable adaptability across diverse climatic zones mainly in Sub-Saharan Africa [125,126], parts of South America, and certain regions of Asia where maize serves as a dietary staple [119].

Furthermore, the co-occurrence of multiple mycotoxins in crops has also been reported, with studies indicating that 30–80% of contaminated samples contain two or more mycotoxin types simultaneously [118]. This multi-contamination phenomenon is particularly pronounced in maize, wheat, and barley, where up to five different mycotoxins may be detected concurrently. The synergistic and additive effects of co-occurring mycotoxins pose complex challenges for risk assessment and regulatory management, as interactive toxicological effects may exceed the sum of individual mycotoxin toxicities [127].

In legumes, contamination is primarily associated with fungi of the genera *Alternaria* and *Fusarium*. A survey of legumes in Russia [128] detected *Alternaria* DNA in 100% of samples and *Fusarium* DNA in 51%, with alternariol occurring in all samples and other toxins such as emodin, deoxynivalenol (DON), diacetoxyscirpenol, and T-2/HT-2 toxins present in 32–42% of samples. Similarly, naturally infected navy beans were found to accumulate diacetoxyscirpenol, DON, T-2 toxin, and fumonisin B_1_, with toxins concentrated in discoloured or damaged seeds [129]. These observations indicate that mycotoxin prevalence in Fabaceae is high and may be underestimated if visual or gross inspection alone is used.

Maize exhibits a similarly high prevalence of mycotoxins, reflecting its global importance as a staple crop and its susceptibility to fungal colonization. Surveys in Ethiopia revealed that freshly harvested maize contained zearalenone in 74% of samples, nivalenol in 63%, DON and DON-3-glucoside in 32%, and multiple fumonisins in 16–41% of samples, with concentrations sometimes exceeding European Union regulatory limits. Post-harvest studies in the same region reported DON and zearalenone in 85% and 81% of samples, respectively, demonstrating that storage conditions can significantly influence toxin prevalence.

The high prevalence of mycotoxins in both Fabaceae and maize underscores the need for integrated management strategies. Preventive measures such as pre- and post-harvest sanitation, sorting and removing discoloured or damaged seeds, cultivation of resistant crop varieties, and rapid detection methods (e.g., qPCR and ELISA) are critical to reduce the risk of contamination. Additionally, agronomic practices including optimized fertilization, pest management, and proper drying and storage—play a pivotal role in mitigating mycotoxin prevalence. The data collectively highlights that mycotoxin contamination is not sporadic but a pervasive challenge in both legumes and maize, necessitating comprehensive monitoring and management across the food value chain [130,131,132].

## 4. Consequences on Human and Animal Health

**Figure 8:**
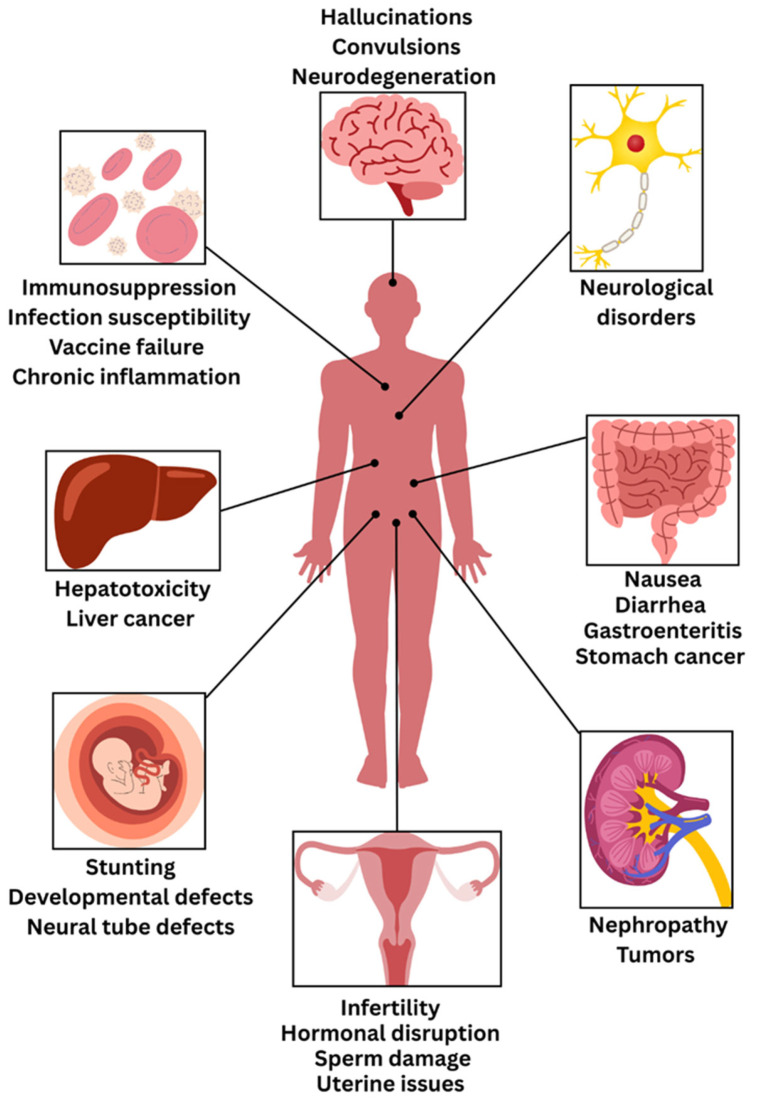
Health Impacts of Mycotoxins.

Mycotoxins cause a wide range of health effects in humans, from acute poisoning to chronic diseases (Figure 8, Table 1). These disease conditions result from exposure to mycotoxins through ingestion, inhalation, or skin contact [22]. Acute mycotoxicoses result from exposure to high doses of mycotoxins over a short period. Symptoms vary depending on the mycotoxin involved but may include gastrointestinal distress, hemorrhage, neurological dysfunction, and, in severe cases, death. Notable examples include aflatoxicosis outbreaks in Western India in 1974, Malaysia in 1988, Kenya in 2004 [22] and trichothecene poisoning (alimentary toxic aleukia) in Russia [133].

Mycotoxins are one of the most significant global food safety concerns and cause a broad range of negative health effects, including acute poisoning, chronic diseases and even death [134]. Importantly, these toxins are reported to contaminate approximately 25% of the world’s food supply, including cereals, nuts, spices, dried fruits, and coffee beans, due to their remarkable chemical stability, which allows them to survive food processing under certain warm and humid conditions [135,136,137]. Most mycotoxins are lipophilic, with notable exceptions including fumonisins which are water-soluble due to their polar tricarballylic acid side chains [34,78]. These toxins can be absorbed by the gastrointestinal and respiratory tracts and distributed via the bloodstream to organs where they exert toxicity, such as the liver, kidneys, and reproductive organs [138]. Current estimates and research show that developing countries face a disproportionately higher mycotoxin burden, with imported data showing contamination in up to 40% of crops grown in developing countries within sub-Saharan Africa. Moreover, an estimated 4.5 billion people worldwide are under chronic mycotoxin exposure. There is an urgent need for a holistic understanding of mycotoxins and their potential threat to health [139].

At the cellular level, mycotoxins exhibit profound genotoxicity by entering human cells and accessing the cellular genome, causing major mutagenic effects, including DNA single-strand breaks, chromosomal aberrations, and DNA fragmentation that can ultimately lead to apoptosis and carcinogenesis [140]. Moreover, the International Agency for Research on Cancer has classified many mycotoxins based on their carcinogenicity, with AFB1 classified as a Group 1 cancer-causing toxin that has a toxicity level 68-fold greater than arsenic [141], causing approximately half of hepatocellular carcinomas in the world by mutating key P53 tumour-suppressing genes [13]. Mycotoxins also have different cancer-causing profiles when analyzed (i.e., different mycotoxin classes can have different carcinogenic characteristics). For example, OTA is a nephrocarcinogen (Group 2B) associated with urinary tract tumours [64], although the condition once linked to OTA, Balkan endemic nephropathy [69], is now attributed to chronic exposure to aristolochic acid [142]. OTA has also been hypothesized to have possible effects on breast and testicular carcinogenesis [31]. Fumonisins, classified as probably carcinogenic (Group 2B), have robust correlations in humans for esophageal cancer and with disrupting the uptake of folic acid into infants, causing neural tube defects, whereas patulin has mutagenic and teratogenic features, causing intestinal and stomach cancer by causing direct cellular harm [137]. Additionally, zearalenone induces mammary tumours and hepatocellular adenomas in animal models with hypothetical implications for liver, kidney, brain, and reproductive organ cancers in humans, while trichothecenes, including T-2 toxin and nivalenol, demonstrate genotoxic potential that increases mutation frequency and cancer risk [27,140,143].

The organ-specific toxicity induced by mycotoxins has numerous manifestations through various pathophysiological mechanisms involving numerous body systems to varying degrees of severity and clinical presentation. The chronic health effects may include nephrotoxicity, immunotoxicity, neurotoxicity, as well as adverse effects on reproduction and growth [139]. Hepatotoxicity is the most prominent concern, where AFs and OTA cause acute liver damage and chronic liver disease. Notably, AFB1 is a potent hepatocarcinogen associated with increased liver cancer incidence, especially in sub-Saharan Africa and Southeast Asia. Its chronic exposure can lead to hepatocellular carcinoma and growth impairment in children [144,145]. Nephrotoxicity, which results from exposure to OTA, AFs and citrinin, a mycotoxin beyond the scope of this review, can lead to kidney damage, progressive nephropathy, and other toxin-associated conditions historically described as yellow rice disease [140]. Gastrointestinal symptoms can manifest due to deoxynivalenol and other trichothecenes, with severe symptoms that include nausea, vomiting, abdominal pain, diarrhea and gastroenteritis; similarly, patulin induces gastrointestinal toxicity by damaging the gut epithelium through covalent interaction with cellular enzymes [31]. Fumonisins are reported to correlate with esophageal cancer, particularly in populations consuming contaminated maize [146]. Moreover, trichothecenes, including deoxynivalenol, are reported to cause gastrointestinal disorders [147]. Reproductive toxicity includes endocrine disruption with zearalenone having strong estrogenic effects resulting in hyperestrogenism and infertility; similarly, zearalenone acts on male reproduction through the poor viability of sperm and poor DNA integrity; and the most severe consequence of ergot alkaloids can cause abortion, with exposure of livestock such as ewes, cattle, pigs, and sheep resulting in similar reproductive toxicities [22].

There is also strong immunosuppressive toxicity shown through multiple mechanisms of action including; AFs suppressing macrophage function, T-cell activation and induce oxidative stress, ochratoxins which adversely impair B-cell function and anti-body production leading to atrophy of immune organs and inflammation, deoxynivalenol which induces protein kinase pathways causing inflammation and produces pro-inflammatory cytokines, and fumonisins which suppress and promote malfunction in lymphocyte proliferation and affects cytokine production [137]. Trichothecenes are also reported to cause immunosuppression [22,147,148]. Thus, immunocompromised patients, such as HIV patients, experience significantly worsened health outcomes when exposed to mycotoxins due to their already compromised immune systems, among other vulnerable populations [149].

Pregnant women and unborn babies are particularly vulnerable populations to mycotoxin exposure, with significant implications for maternal and fetal health outcomes [150]. Maternal AF exposure has been strongly associated with fetal growth restriction and reduced birth weight, with some studies noting stronger effects in female neonates as the mycotoxins from maternally contaminated food can cross the placental barrier and affect developing fetal systems [37,150]. Fumonisins pose additional developmental risks by increasing the likelihood of neural tube defects through the disruption of sphingolipid biosynthesis, which is critical for folate metabolism [150,151]. Meanwhile, OTA has been detected in cord blood, suggesting prenatal transfer that may further depress birth weight when combined with AF exposure [150,152].

Infants and young children face continued vulnerability through multiple exposure pathways during critical developmental periods. Mycotoxins can be transferred postnatally through breast milk, particularly aflatoxin M1 and ochratoxin A, with levels in many regions exceeding established safety limits [150]. Contamination has also been documented in infant formulas and baby foods, especially cereal-based products commonly consumed during the weaning period [153]. Even low levels of mycotoxin can result in decreased growth, developmental impairments, and increased levels of toxicological sensitivity owing to some combination of continuing physiological development as well as the health of psychological systems in general [136]. Early childhood exposure to AFs and fumonisins demonstrates clear associations with stunting and underweight conditions in children, while trichothecenes are linked to vomiting episodes and growth retardation [150]. Moreover, OTA and sterigmatocystin have been implicated in neurodevelopmental concerns, including autism spectrum disorders and attention deficit disorders in exposed children, highlighting the critical importance of mycotoxin prevention during early life stages [154].

Additionally, mycotoxins exhibit synergistic toxic effects when co-occurring, amplifying the health risks, emphasizing the urgent need for comprehensive food safety strategies [155].

**Table 1:** Major Mycotoxins: Food Sources, Human and Animal Health Impacts.

Mycotoxin	Food Commodity	Impact on Humans	Impact on Animals	References
Aflatoxins	Wheat, walnut, maize, peanuts, eggs, milk, meat	Hepatotoxicity, teratogenicity, carcinogenicity, immunotoxicity	Immunosuppression, productivity reduction, appetite loss, organ damage (Liver, Kidney)	[38,156,157]
Ochratoxins	Coffee beans, oats, wheat, maize, wine, dried fruits, spices	Nephrotoxicity, hepatotoxicity, genotoxicity, neurotoxicity	Nephrotoxicity, reduction in growth rate, Immunosuppression, poor performance	[158,159,160]
Fumonisins	Corn flour, peanut, grapes, rice, wheat, barley,	Esophageal cancer, liver cancer, neural tube defects, child growth defects	Porcine pulmonary edema, Equine leucoencephalomalacia, skeletal abnormalities	[19,161,162]
Trichothecenes	Barley, oats, wheat, maize	Skin irritation, gastrointestinal distress, alimentary toxic aleukia, respiratory issues	Feed refusal, weight loss, immunosuppression, dermatitis	[163,164]
Zearalenone	Wheat, barley, sorghum, rye, rice	Reproductive system disorders, hepatotoxicity	Immunotoxicity, reproductive system defects, hormonal defects	[165,166,167]
Patulin	Apple, fig, tomatoes, grapes,	Gastrointestinal issues, nausea, vomiting	Teratogenicity, organ damage (kidney, liver), immune system toxicity, brain edema	[168,169,170]

## 5. Mycotoxin Control and Prevention

An integrative approach that combines agronomic, biological, chemical, and regulatory measures is essential for the proficient management and mitigation of risks associated with mycotoxins. Continued research and advancements in detection methods, detoxification processes, and regulatory systems will be vital in mitigating the adverse effects of mycotoxins and in securing a more reliable food supply worldwide.

### 5.1. Pre-Harvest Control Measures

Control strategies in the pre-harvest stage are essential for mitigating fungal infestations and the subsequent production of mycotoxins [171]. Effective pre-harvest suppression of mycotoxins hinges on lowering plant stress, blocking insect-mediated injury, and shifting the field microbiome away from toxigenic fungi—using genetics, biocontrol, and agronomy as an integrative approach. Implementing appropriate agronomic techniques, including crop rotation, effective irrigation management, and timely harvesting, serves to significantly reduce fungal proliferation within agricultural fields [172]. The advancement and implementation of mycotoxin-resistant agricultural varieties, achieved through conventional breeding techniques [173,174] and genetic modification, significantly contribute to the reduction in contamination levels [175]. Insect-mediated injury is a primary infection court for *Fusarium* and *Aspergillus*; therefore, deploying insect-resistant *Bacillus thuringiensis* (Bt) hybrids and robust Integrated Pest Management for ear-feeding Lepidoptera yields direct toxin benefits. A continent-scale analysis showed Bt maize had ~29% lower total mycotoxins, including ~30.6% fumonisins and ~36.5% trichothecenes; newer reviews similarly conclude consistent reductions in fumonisins and aflatoxins in Bt events [176]. According to Mesterhazy et al. (2022) [177], beyond Bt traits, host genetics play a critical role in reducing mycotoxin risk. Selecting maize hybrids with proven resistance to ear rot and, in some regions, using aflatoxin-resistant germplasm such as Mp-derived lines, which are maize inbred lines developed by the USDA Agricultural Research Service (ARS) specifically for aflatoxin resistance, can significantly lower field contamination and provide more stable performance across seasons. Recently developed methods for ranking hybrids based on food-safety risk should also be integrated into variety selection guides. Biological control strategies, encompassing the utilization of non-toxigenic fungal variants such as *A*. *flavus* AF36 [178,179], have been effectively implemented to surpass toxin-generating fungal species [180]. For aflatoxins (AFB1) in maize and groundnut (Fabaceae), atoxigenic *A*. *flavus* biocontrol routinely cuts field and storage contamination by ≥80% in multi-year programmes, while large private-sector datasets report treated maize frequently <4 µg kg^−1^ while paired untreated lots averaged 7.6–38.5 µg kg^−1^ (8.7–97.4% reductions across sites). Comparable efficacy has been demonstrated in groundnut/peanut as well as maize in West and East Africa, underscoring transferability to Fabaceae systems [181]. Fungicides are used extensively in agricultural systems to manage diseases and preserve crop yield and quality [182,183]. Although fungicides and antifungal agents can limit fungal growth, careful monitoring is essential in preventing chemical residues in food products [184]. Identification of high-risk conditions and implementing timely interventions are assisted by environmental monitoring and predictive models [185]. To maximize fungicide application schedules and attain high efficacy, it is essential to accurately estimate disease prevalence, onset timing, and progression. Machine learning (ML) models have been used in studies to help farmers make accurate choices about managing [186,187]. Managing agronomic factors that reduce drought and heat stress are major drivers of aflatoxin contamination and have proven highly effective. Field trials and extension demonstrations have shown that supplemental irrigation during grain filling can reduce aflatoxin levels by about 70%. In addition, drought indices and mechanistic models such as AFLA-maize now enable in-season risk forecasting and more precise intervention timing [188]. The planting date is another critical factor: early or optimally timed sowing consistently reduces Fusarium ear rot severity and fumonisin B_1_ levels, with multiple studies across North America and Europe confirming significantly lower contamination in earlier-planted maize [189].

### 5.2. Post-Harvest Control Measures

Mycotoxin accumulates in stored commodities. To be effective, these measures must focus on rapid moisture reduction, controlled storage environments, physical removal of contaminated grains, and decontamination strategies. Post-harvest control measures primarily aim to inhibit the proliferation of fungi and the subsequent accumulation of mycotoxins in stored agricultural products [185,186]. Optimal storage conditions, which include the maintenance of moisture levels below 13% and the utilization of hermetic (airtight) storage systems, have been shown to significantly diminish fungal contamination [189,190,191,192]. As examples storage fungi (e.g., Aspergillus, *Penicillium*) can grow at moisture levels as low as 13%, with *A*. *flavus* growing above 18% moisture [193]. Achieving and maintaining grain moisture below 13% is vital because at these levels, mycotoxin-producing fungi are typically unable to thrive. According to Wyllie [193], quick high-temperature drying is more effective than slow, low-temperature options in halting fungal growth, even though it cannot remove existing toxins. Grain drying generally reduces moisture from ~17–30% to safe storage levels between 8–15%, depending on the crop type. Hermetic storage is exceptionally effective at limiting both fungal growth and mycotoxin buildup without using chemical preservatives. In a year-long study of smallholder farms by Dembedza et al. [194], the percentage of samples showing aflatoxin M_1_ rose from 5.4% at harvest to 75% nine months later under conventional storage. In contrast, hermetic storage reduced both the detection frequency (by up to 33.2%) and toxin levels (by up to 48.7%). Comparative trials in Kenya found that hermetic devices (i.e., metal silos) limited aflatoxin increases to less than 5% per month, whereas polypropylene bags showed a strong correlation between moisture, time, and rising aflatoxin levels [195]. In Zimbabwe, maize stored in hermetic containers had significantly lower aflatoxin B_1_ levels than those in conventional storage, while fumonisin B_1_ increases were similar across both storage types [196]. Sorting and physical removal of contaminated grains is essential in reducing the risk of mycotoxin exposure, as these toxic compounds can lead to serious health issues in both humans and animals [13,196]. Effective screening aids in preventing high-toxin kernels from entering the food or feed supply chain. Decontamination techniques (chemical, physical, and biological methods) also play a key role in safeguarding food safety, helping to remove or reduce mycotoxin levels in affected products before they reach consumers [13,197,198].

### 5.3. Mycotoxin Detoxification Strategies

Detoxification of mycotoxins is a critical component of food and feed safety, especially given the persistence and stability of these compounds under standard processing conditions. Strategies can be broadly categorized into physical, chemical, biological, and innovative processing-based approaches, each with distinct mechanisms and efficacy.

#### 5.3.1. Physical Detoxification Approaches

The implementation of heat treatment, ultraviolet radiation, high-pressure processing (HPP) and irradiation has demonstrated significant reductions in mycotoxin concentrations, although their effectiveness varies by toxin type and matrix [140,197,199]. For example, aflatoxins are moderately heat-labile, with reductions of up to 70–90% during extrusion cooking at temperatures above 150 °C, whereas trichothecenes such as DON are highly heat-stable, often requiring combined physical–chemical strategies for effective degradation [197,198]. Ultraviolet radiation has been particularly effective against aflatoxin B_1_, with reductions exceeding 80% after 30–60 min of UV exposure, depending on grain moisture content [199]. Gamma and electron-beam irradiation are also widely tested, achieving up to 95% toxin reduction under optimized conditions while maintaining product quality [140]. HPP has shown substantial decreases in toxins such as those produced by *Fusarium graminearum*, yet still underexplored, particularly regarding the identity and safety of the compounds formed during treatment [22]. Structural modifications generated during these treatments may also result in masked mycotoxins when toxins interact with plant macromolecules [22,31], an issue that has become a key focus of current mycotoxin research.

#### 5.3.2. Chemical Detoxification Approaches

Chemical methods rely on reactive agents to degrade mycotoxins into less toxic derivatives. Chemical detoxification methodologies, including ammoniation and ozonation, have been utilized to decompose mycotoxins [200,201,202]. Ammoniation, widely applied in the detoxification of aflatoxin-contaminated maize and groundnuts, has been reported to reduce toxin concentrations by more than 95%, with approval from regulators in several countries for feed treatment [200]. Ozonation has gained prominence as a safer alternative, with studies demonstrating the complete degradation of aflatoxin B_1_ within minutes of exposure to 60 mg/L ozone [201]. Recent work has also highlighted the use of food-grade oxidants, acids, and alkaline agents to degrade fumonisins and zearalenone, though optimization is required to prevent negative impacts on nutritional quality [202].

#### 5.3.3. Adsorbent-Based Detoxification

The inclusion of adsorbents in animal feed is among the most widely adopted strategies to reduce the bioavailability of ingested mycotoxins. Activated carbon, hydrated sodium calcium aluminosilicate (HSCAS), and bentonite clays have shown binding efficiencies ranging from 60–95% for aflatoxins under gastrointestinal conditions [203,204]. However, adsorption efficiency depends strongly on the polarity and structure of the toxin. While aflatoxins are efficiently sequestered, fusariotoxins such as DON and T-2 toxin are less effectively bound. Similarly, chitosan exhibited markedly lower binding activity against AFB1, FB1, OTA, T-2, DON, and ZEA compared to cellulosic polymers, while activated carbon successfully removed aflatoxins and patulin from contaminated milk and cider, respectively [31,204]. Furthermore, aluminosilicates and activated carbon may indiscriminately sequester micronutrients and vitamins through non-selective adsorption, necessitating careful dosage optimization to prevent nutrient depletion and maintain feed quality [22,31]. Additionally, adsorbent efficacy is frequently compromised under conditions of elevated mycotoxin concentrations or when multiple toxins co-occur, as several mycotoxins simultaneously contaminate feeds in nature [31]. These constraints underscore the necessity for integrated mycotoxin management strategies combining adsorption with complementary detoxification approaches to ensure optimal livestock health and production performance.5.3.4. Biological detoxification approaches

Biological detoxification strategies, encompassing enzymatic degradation and microbial treatments involving lactic acid bacteria and yeasts, present an environmentally sustainable alternative for reducing mycotoxins [31,205,206]. Microbial degradation using lactic acid bacteria (LAB), yeasts (*Saccharomyces cerevisiae*), and filamentous fungi has been shown to enzymatically convert mycotoxins into less toxic or non-toxic metabolites [31,205]. For example, certain LAB strains metabolize aflatoxin B_1_ into aflatoxicol, which exhibits markedly reduced toxicity. Similarly, zearalenone-degrading enzymes from Bacillus species can cleave the lactone ring, reducing estrogenic activity [31]. The role of gut microbiota is also increasingly recognized, with commensal bacteria and archaea in the gastrointestinal tract shown to immobilize or enzymatically biotransform toxins such as DON and fumonisins into metabolites of diminished toxicity [207]. While microbial degradation is generally milder than physical or chemical treatments and better preserves nutritional quality [200,201], it can result in unintended substrate metabolism, potentially reducing raw material quality [31]. Enzymatic degradation provides enhanced substrate specificity; nevertheless, off-target catalytic activity toward non-target food components remains a concern, as certain mycotoxin-degrading enzymes may react with other molecules present in complex food matrices [31,200]5.3.5. Innovative processing technologies

Modern food-processing technologies are also highly effective in mitigating mycotoxin contamination. High-pressure processing (HPP) disrupts toxin stability through pressure-induced conformational changes, achieving up to 60% reductions in zearalenone and aflatoxins in certain matrices [208]. Extrusion cooking, by combining high temperature, shear force, and moisture, not only degrades heat-labile toxins but also enhances adsorption when combined with binders [209]. Fermentation processes, particularly with starter cultures in cereals and dairy products, can reduce mycotoxin levels by 40–80%, as microbial enzymes degrade toxins or bind them irreversibly to cell wall components [210].

### 5.4. Regulatory Frameworks and Risk Assessment

Regulatory frameworks and monitoring methodologies are vital for safeguarding food safety and reducing exposure to mycotoxins [211]. Before the end of the 1990s, the establishment of mycotoxin regulations predominantly occurred at a national level. Over time, various economic coalitions such as the European Union, MERCOSUR, and the jurisdictions of Australia and New Zealand have aligned their mycotoxin regulatory frameworks, superseding existing national laws. Contemporary regulations are increasingly predicated upon scientific assessments from esteemed organizations, such as the FAO/WHO Joint Expert Committee on Food Additives of the United Nations (JECFA) and the European Food Safety Authority (EFSA) [212]. National and international entities, such as the Food and Agriculture Organization (FAO) and the World Health Organization (WHO), establish maximum allowable thresholds for mycotoxins in food and feed commodities to safeguard public health [213]. Promoting collaborations and cooperative research initiatives among scientists and laboratories within Asian nations is essential to evaluate the magnitude of human exposure to mycotoxins in this region [214]. Sri Lanka is presently engaged in a concerted effort in partnership with the Food and Agriculture Organisation (FAO) concerning the ongoing harmonization of food regulations within the South Asian Association for Regional Cooperation (SAARC) region. In pursuit of fortifying its food regulatory framework, Sri Lanka has initiated a comprehensive review of its existing food regulations, aligning them with the directives set forth by the Codex Alimentarius Commission, facilitated by the expertise of local consultants [215]. Advanced analytical methodologies, including high-performance liquid chromatography (HPLC), enzyme-linked immunosorbent assay (ELISA), and liquid chromatography-mass spectrometry (LC-MS), facilitate precise identification and quantification of mycotoxins in food matrices [13,216,217,218]. Initiatives aimed at increasing consumer awareness and educational outreach are also crucial in fostering optimal practices for the prevention of mycotoxins among agricultural producers, food manufacturers, and consumers [219,220].

## 6. Emerging Detection Methods of Mycotoxins in Food

Mycotoxins represent one of the most pervasive and economically devastating food safety threats facing modern agriculture. These toxic secondary metabolites contaminate approximately 25% of the world’s crops annually, resulting in the loss of roughly 1 billion metric tons of food each year [45,221]. The human toll is staggering, with an estimated 500 million people in developing nations directly affected by mycotoxin exposure, while even affluent urban populations remain vulnerable to contamination risks in densely populated environments where favourable conditions promote mould growth and toxin production [222].

The economic consequences extend far beyond immediate crop losses. European wheat alone incurred EUR 3 billion in losses from deoxynivalenol contamination between 2010–2019, with aflatoxin contamination causing an additional EUR 2.5 billion in economic damage [186,223]. These figures only reflect documented losses in regulated markets and likely underestimate the true global impact, especially in regions with limited monitoring infrastructure.

The fundamental challenge lies in the inevitable nature of mycotoxin contamination. As natural food contaminants cannot be eradicated from agricultural systems, mycotoxins demand continuous vigilance through sophisticated detection strategies [224]. Regulatory frameworks worldwide have established maximum permissible levels, but enforcement depends critically on the availability of reliable, accessible, and cost-effective detection technologies that can operate across diverse agricultural and processing environments.

**Figure 9:**
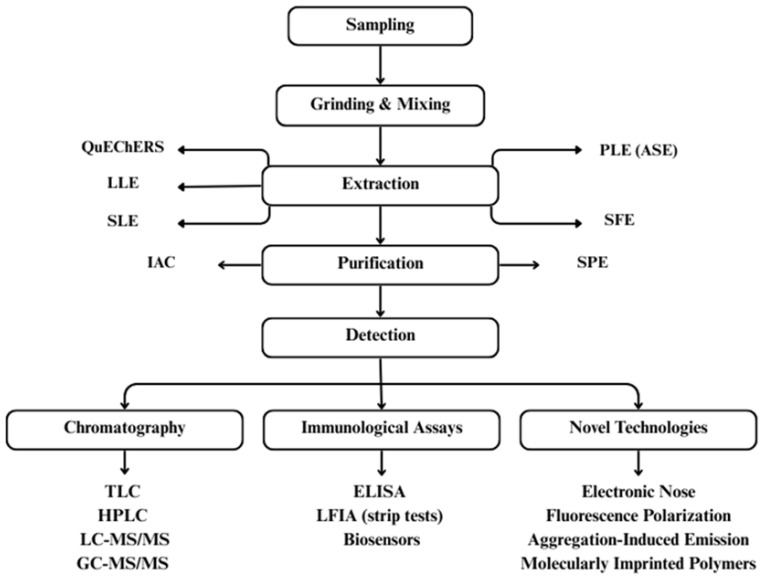
Workflow for mycotoxin detection. The process includes sampling, grinding, and mixing, followed by extraction using QuEChERS (Quick, Easy, Cheap, Effective, Rugged, Safe), LLE (Liquid–Liquid Extraction), SLE (Solid–Liquid Extraction), PLE/ASE (Pressurized/Accelerated Solvent Extraction), or SFE (Supercritical Fluid Extraction). Purification is performed with IAC (Immunoaffinity Columns) or SPE (Solid Phase Extraction), and detection is achieved through chromatography [TLC (Thin Layer Chromatography), HPLC (High-Performance Liquid Chromatography), LC-MS/MS (Liquid Chromatography–Tandem Mass Spectrometry), GC-MS/MS (Gas Chromatography–Tandem Mass Spectrometry)], rapid methods [ELISA (Enzyme-Linked Immunosorbent Assay), LFIA (Lateral Flow Immunoassay), biosensors], or novel technologies (electronic nose, fluorescence polarization, aggregation-induced emission, molecularly imprinted polymers).

Current detection standards face a significant mismatch between technological capabilities and real-world needs. The standardized procedure for mycotoxin detection is outlined in Figure 9. Traditional chromatographic and immunoassay methods offer exceptional accuracy but demand specialized infrastructure, skilled personnel, and significant time investments that render them impractical for routine monitoring in many settings.

This technological gap has driven intensive research into artificial intelligence-driven approaches that promise to democratize mycotoxin detection while maintaining analytical rigour.

The emergence of artificial intelligence (AI) and machine learning (ML) technologies in mycotoxin detection represents a transformative approach to addressing one of the most pressing challenges in global food safety. Machine learning methods have gained popularity for mycotoxin detection due to their accurate and timely predictions, offering solutions to many limitations of traditional approaches over the past decade [225].

### 6.1. Artificial Intelligence and Machine Learning

Artificial Intelligence algorithms such as support vector machines (SVMs), artificial neural networks (ANNs), and random forests (RFs), have been widely tested across food matrices with encouraging results. In peanuts, ANN-based models achieved correlation coefficients of 0.91 for aflatoxin B1 detection using olfactory visualization and nearly 100% accuracy in detecting fungal development under LED imaging [223]. In wheat, RFs trained on phenological, and meteorological data reached 99% accuracy in internal validation for multi-mycotoxin prediction, though external validation dropped to 90%, highlighting problems of overfitting and poor generalization [223,225].

Deep learning approaches, especially convolutional neural networks (CNNs), have further improved detection accuracy. CNNs achieved 100% accuracy for aflatoxin detection in wheat using microwave imaging and 97% precision in identifying Fusarium head blight from RGB images. More sophisticated architectures, including GoogLeNet-CNN and transformer-based models, have exceeded 96% accuracy in cocoa and peanut aflatoxin detection [223]. However, the “black box” nature of deep learning models presents significant interpretability challenges that may hinder regulatory acceptance. The complexity of these models makes it difficult to understand decision-making processes, potentially limiting stakeholder confidence in AI-based systems. This lack of transparency poses substantial barriers to regulatory approval and widespread adoption in food safety applications where explainability is crucial for validation and trust [218].

### 6.2. Integration with Advanced Sensing Technologies

#### 6.2.1. Hyperspectral Imaging

Hyperspectral Imaging (HSI) is regarded as the most promising and powerful imaging approach for mycotoxin detection using ML, as it can describe the chemical properties of agricultural foods and food products. The combination of hyperspectral imaging with ML has yielded impressive results across various crops. For maize analysis, sparse auto-encoder and CNN integration with SVM classification achieved 99.47% accuracy in mouldy kernel detection [223]. Studies on maize and peanuts have shown that HSI can accurately differentiate contaminated kernels, sometimes down to individual toxins such as aflatoxin B1 [226,227]. Its non-destructive nature makes it particularly appealing for rapid, large-scale screening in grain supply chains [228].

The non-destructive nature of hyperspectral imaging offers a significant advantage over traditional analytical methods, allowing for real-time quality assessment without damaging the sample [229]. Recent research has focused on developing accurate models for on-site mycotoxin detection, particularly in grains and oilseeds, including maize, wheat, and peanuts, which are vulnerable to fungi and mycotoxin contamination [228]. Technology’s ability to capture both spatial and spectral information enables precise localization of contamination within individual kernels or food matrices, facilitating targeted quality control interventions [226].

#### 6.2.2. Electronic Nose Technology

Electronic nose (e-nose) systems coupled with AI algorithms offer promising solutions for rapid, non-destructive mycotoxin detection through volatile compound analysis. It is designed to mimic human olfactory detection through sensor arrays that respond to volatile organic compounds emitted by fungal metabolism. This approach is attractive for its speed and low cost, particularly in detecting spoilage before toxins reach harmful levels [230,231,232].

Cheli et al. [231] argue that while e-nose systems have demonstrated strong laboratory performance, their stability in field conditions remains questionable. Fluctuations in humidity, temperature, and the presence of non-fungal volatiles frequently compromise accuracy. Unlike HSI or biosensors, which can provide quantitative toxin data, e-nose technology is limited to indirect detection of fungal activity, making it better suited as a supplementary tool rather than a standalone solution.

In a study by Leggieri et al. [233], an AIR PEN 3 portable e-nose system achieved 83% accuracy for deoxynivalenol detection in wheat, while achieving 96.4% accuracy for *Aspergillus* species detection in rice using back propagation neural networks. E-nose has high discrimination accuracy between non-contaminated and single-mycotoxin-contaminated grain; however, predictive accuracy remains limited and unsuitable for in-field application where mycotoxin co-contamination occurs.

#### 6.2.3. Biosensor Technologies

Advanced biosensors incorporating miniaturized metres are capable of accurate, quantitative, and real-time analysis for point-of-care testing [234]. The integration of miniaturized metres into advanced biosensors makes them ideally appropriate for portable, on-site mycotoxin detection [235]. Recent advances in nanobiosensors demonstrate increased sensitivity compared to conventional methods. The use of nanostructured materials such as gold nanoparticles, carbon nanotubes, and quantum dots improves detection limits and specificity by amplifying signal responses [236,237]. Electrochemiluminescence biosensors have gained attention for mycotoxin detection due to their high sensitivity and specificity, with recognition mechanisms mainly divided into antibody-based and aptamer-based approaches. Gold nanoparticle-based apta sensors targeting multiple analytes, including mycotoxins, showed superior performance to standardized detection methods [223]. Nanoengineered optical biosensors provide high sensing performance and fast, accurate bio-detection screening, which is attractive for industrial applications [235]. These miniaturized systems offer the potential for democratizing mycotoxin detection, making sophisticated analytical capabilities accessible to resource-limited settings and enabling distributed monitoring networks.

However, most biosensors remain in the experimental stage, with limited validation in real-world food matrices where multiple mycotoxins often coexist. Cross-reactivity and matrix interference remain serious obstacles, especially in commodities like cereals and nuts, where protein, fat, and fibre content can obscure detection signals [217]. The evolution of biosensor technologies toward increased sensitivity, selectivity, and miniaturization positions them as critical components in next-generation food safety monitoring systems. However, challenges related to sensor stability, standardization, and regulatory validation must be addressed to maximize their potential in practical applications.

## 7. Emerging Detection Methods of Mycotoxins in Food

Despite decades of research, new challenges such as climate change, technological advances, and global trade are reshaping both the risk landscape and the effectiveness of management strategies.

### 7.1. Climate Change and Mycotoxin Contamination Patterns

One of the most urgent emerging challenges in mycotoxin research is the impact of climate change on contamination patterns. Rising global temperatures and shifting weather conditions are altering the distribution and population dynamics of crop pests [238]. This indirectly aggravates mycotoxin contamination, as insect damage provides entry points for fungi, facilitating colonization and toxin production. At the same time, climate change is expected to drive crop production into cooler regions, reshaping crop distribution and exposing new plant–pathogen interactions. This shift may also change the prevalence of mycotoxin-producing fungal species, leading to previously unrecognized risks [188,239]. Such geographic redistribution of contamination risk zones poses significant challenges for food safety systems, many of which are still designed around historical contamination patterns. The problem is further complicated by the dynamic nature of fungal populations and the diversity of toxins they produce, with overall contamination levels projected to increase as climate pressures intensify. While substantial research has explored the effects of climate change on AFs, deoxynivalenol, and OTA [238], a fewer number of studies have examined its impact on fumonisins and alternaria toxins. This knowledge gap is particularly concerning given the widespread occurrence of these mycotoxins in staple crops, and their serious health consequences [22]. Addressing this issue will require the development of predictive models capable of simulating contamination risks under different climate scenarios. Such models must integrate multiple variables such as temperature, humidity, crop physiology, pest dynamics, and fungal ecology to generate actionable insights. Reliable forecasting tools of this kind would be invaluable for farmers, food processors, and regulatory authorities working to safeguard food supplies amid climate uncertainty.

### 7.2. AI and ML in Mycotoxin Detection

The field of mycotoxin detection is undergoing a technological revolution, with AI and ML emerging as transformative tools. AI has been introduced as a new technique for mycotoxin detection in food, providing high credibility and accuracy, and addressing significant limitations of traditional analytical methods [186,223]. Traditional lab analysis methods for mycotoxin detection can be time-consuming and may not always be suitable for large-scale screenings [218]. Recent developments include promising real-time mycotoxin detection methods based on AI technology that have managed to overcome these limitations, with some systems like ImagoAI offering results in 30 s using advanced artificial intelligence and hyperspectral imaging to quickly and accurately detect multiple mycotoxins in a single analysis [228,240]. These technological advances represent a paradigm shift from laboratory-based analysis to field-deployable, real-time monitoring systems.

Future advances in mycotoxin detection will probably focus on the integrating multiple analytical modalities with AI processing. Combining hyperspectral imaging, electronic nose tech, and biosensors could offer complementary data to improve detection accuracy and lower false positives [231,235]. However, this requires advanced data fusion algorithms and standardized protocols for collecting multi-modal data.

These technological advances have profound implications for global food safety systems. Real-time detection capabilities enable proactive rather than reactive approaches to mycotoxin management, allowing for immediate intervention when contamination is detected. This shift from post-harvest testing to continuous monitoring could dramatically reduce the amount of contaminated food entering the supply chain and minimize consumer exposure [216]. However, the implementation of these advanced detection technologies faces significant challenges, especially in developing regions where mycotoxin contamination is often highest, but technological infrastructure may be limited. Addressing these equity issues will be crucial for realizing the full potential of AI-driven detection systems.

### 7.3. Challenges in AI-Based Detection Implementation

Emerging AI-based methods show promise for real-time mycotoxin detection, overcoming the limitations of traditional approaches [225]. However, a significant gap exists between cutting-edge AI research and the practical needs of food producers, especially in resource-limited settings. Future efforts should focus on creating lightweight, low-power AI models that can operate on mobile devices or affordable hardware. This democratization of technology is crucial for tackling mycotoxin contamination in high-risk, low-resource regions. Furthermore, the regulatory framework for AI-based detection is still underdeveloped [211]. Establishing clear guidelines for validation, benchmarking, and quality assurance of AI systems is essential to encourage industry adoption. Agencies need to develop balanced frameworks that foster innovation while ensuring safety, so AI methods meet strict food safety standards.

The development and implementation of AI-based mycotoxin detection systems face several critical challenges that must be addressed for successful adoption. Data quality and availability represent fundamental limitations, as robust AI models require large, diverse, and well-annotated datasets that capture real-world variability in food matrices, environmental conditions, and contamination patterns, yet current datasets often lack sufficient scope and complexity for comprehensive training [45]. Cross-matrix generalization poses another significant technical hurdle, where models trained on specific food products, such as wheat, frequently fail to perform adequately when applied to different matrices, such as maize or rice, necessitating the development of universal models capable of maintaining accuracy across diverse food systems [227]. Economic viability concerns centre on the substantial initial investments required for hardware, software, and specialized expertise, which must be weighed against long-term cost savings and the value of prevented contamination incidents, particularly challenging small-scale producers who may lack the resources for such investments without appropriate support mechanisms. Finally, ethical and legal considerations encompass complex questions regarding liability distribution, data privacy protection, and decision-making authority, requiring comprehensive regulatory frameworks that address scenarios involving AI system failures, false positive results leading to unnecessary recalls, and the assignment of legal responsibility for AI-driven food safety decisions [223,231,241].

Methodological advancements, collaborative research, and regulatory framework advances are three interconnected areas that require coordinated attention to produce evidence-based recommendations for improving AI-based mycotoxin identification and detection. Methodological improvements must prioritize rigorous experimental design incorporating independent validation datasets, standardized performance metrics, and transparent reporting of model limitations, with cross-validation procedures that include comprehensive testing across diverse environmental conditions and food matrices to ensure robust and reliable performance assessment [224]. Collaborative research initiatives are essential for bridging the gap between academic research and practical implementation, requiring multidisciplinary cooperation among food scientists, computer scientists, regulatory experts, and industry stakeholders to develop validated AI solutions through coordinated efforts that establish shared datasets, standardized protocols, and common evaluation frameworks that can accelerate progress while maintaining quality standards. Regulatory framework development represents a proactive necessity, where agencies must establish comprehensive guidelines for AI-based mycotoxin detection methods that include specific performance benchmarks, validation requirements, and quality assurance protocols, carefully balancing the encouragement of innovation with safety imperatives while providing clear, accessible pathways for technology adoption that enable both large-scale and small-scale food producers to implement these advanced detection systems effectively.

### 7.4. Global Trade and Economic Impact

Mycotoxin contamination creates significant barriers to international trade, particularly affecting developing countries seeking to export agricultural products to markets with strict mycotoxin limits. These trade barriers can spread economic inequalities and limit opportunities for agricultural development in regions where mycotoxin management is most challenging. The economic magnitude of this challenge is staggering, with Wu [30] demonstrating that the annual economic impact of aflatoxin and fumonisin regulation in the United States alone reaches nearly $1 billion, while projections suggest that global costs could potentially exceed $500 billion if regulatory thresholds were universally applied. Recent comprehensive analyses have reinforced these concerns, with global agro-economic industry losses continuing to escalate due to cross-border import-export denials, product destruction, and routine analysis requirements [242], particularly impacting goods from regions accounting for 70% of global nut and dried fruit imports [243]. The evolution of mycotoxin regulations has created a patchwork of standards that reflect economic and technological capacities rather than purely scientific considerations, with the European Union establishing the most stringent regulations through Commission Regulation (EU) 2023/915 and recently updating deoxynivalenol (DON) limits to 1000 ppb for unprocessed cereal grains in 2024 [244].

The regulatory landscape shows major global differences. Mycotoxin regulations vary from country to country, and the level of detail and strictness also varies [214]. This creates an uneven trading environment that tends to benefit countries with stronger monitoring systems. For developing countries, like Sri Lanka, positioned along the equator where environmental conditions naturally promote proliferation of mycotoxigenic fungi, mycotoxin contamination creates a significant challenge [172]. For example, aflatoxins have been detected in a wide range of Sri Lankan staples, including rice, maize, lentils, peanuts, copra, and spices, with several commodities exceeding EU limits [245]. Coconut oil, one of the country’s major exports, illustrates these trade risks: while branded virgin coconut oils destined for export often comply, bulk unbranded copra coconut oils from many small-scale producers do not satisfactorily comply with EU aflatoxin standards, intended for human consumption [246]. Testing capacity has improved through the ISO/IEC 17025 accreditation of the Food Safety & Quality Assurance Laboratory in Peradeniya, but smallholders often lack affordable access to accredited facilities [247], resulting in disproportionately high rejection rates relative to the country’s export share. The consequences extend far beyond immediate health concerns, as strict limits in high-income markets function as de facto trade barriers, forcing exporters to divert products to less stringent markets or lower-value uses, thereby undermining income and growth potential [220]. Addressing these challenges requires harmonized international standards, risk-based approaches, and capacity-building initiatives to ensure that global food safety regulation protects consumers without excluding producers in vulnerable regions from accessing premium markets.

**Figure 10:**
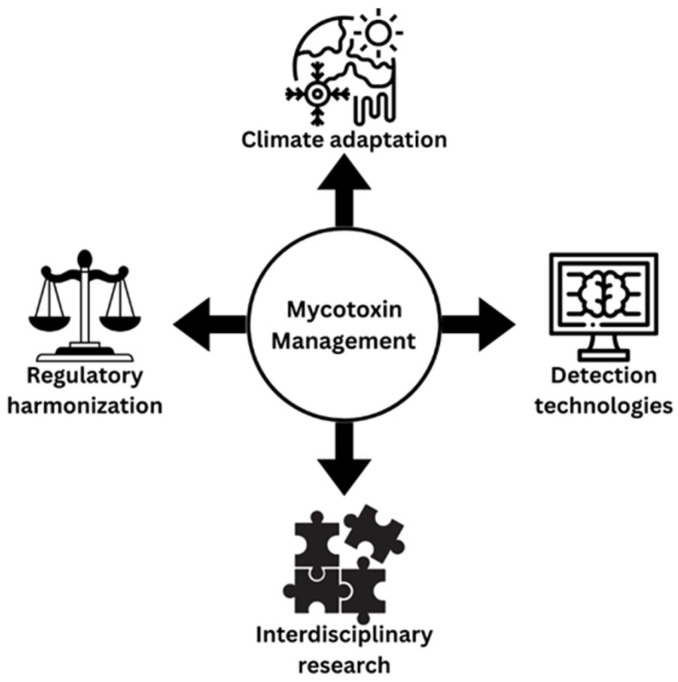
Mycotoxin Contamination Management Framework.

Future research priorities for mycotoxin management highlight critical knowledge gaps and emerging opportunities that demand comprehensive, interdisciplinary approaches to effectively address this complex global challenge (Figure 10). Several urgent research areas require immediate attention, including climate change adaptation through comprehensive modelling of mycotoxin distribution patterns under various climate scenarios that integrate complex interactions between climate variables, crop physiology, fungal ecology, and pest dynamics to develop proactive adaptation strategies [238]. Additionally, research on synergistic effects must investigate the health implications of multi-mycotoxin exposure to establish appropriate risk assessment models and regulatory limits that account for combined effects rather than individual compound assessments [4,139]. Sustainable control technology development represents a critical priority for global food security, requiring environmentally friendly, cost-effective strategies specifically designed for resource-limited settings [187]. This could include the development of precision agriculture strategies that employ machine learning and IoT applications for predictive modelling, and for precision delivery of control measures [187]. This is likely to revolutionize mycotoxin management by allowing real-time monitoring of conditions that host mycotoxigenic moulds and their targeted interventions. The complexity of mycotoxin contamination as a “wicked problem” characterized by multiple stakeholders, competing objectives, and interconnected systems highlights the need for interdisciplinary approaches that combine fungal biology, agricultural science, food technology, public health, economics, and policy research to create holistic solutions that address technical, economic, and social dimensions simultaneously [219].

## 8. Conclusions

With over 400 documented mycotoxins worldwide, the potential for approximately 4.5 billion people to be chronically exposed to mycotoxins should not be underestimated. The six main classes of these toxic compounds are: aflatoxins, ochratoxins, trichothecenes, fumonisins, zearalenone, and patulin. They continue to dominate research focus, given their global presence in staple food crops and notable health impacts on humans and animals. To manage mycotoxins effectively, a comprehensive, multifaceted approach is needed, employing all pre- and post-harvest interventions, and a well-defined regulatory strategy. However, this approach demands innovative technological solutions, as the traditional detection methods prove inadequate for the scale and urgency of mycotoxin contamination, affecting 25% of the world’s crops and 500 million people annually. Innovative solutions include the integration of artificial intelligence, machine learning, and advanced sensing technologies to develop accessible real-time detection systems that monitor and control mycotoxin risks across diverse agricultural environments worldwide. While AI and real-time monitoring offer promising solutions, barriers such as data quality, cost, and regulatory gaps remain. In addition, mycotoxin management faces growing challenges from climate change, uneven regulations, and limited detection capacity in high-risk regions. Future efforts must focus on predictive modelling, accessible detection tools, standardized regulations, and interdisciplinary collaboration to safeguard food security in a changing global landscape. Achieving safe and equitable food systems in the face of mycotoxin challenges will depend on uniting scientific innovation, practical solutions, and global cooperation.

## Data Availability

No new data were created or analyzed in this study. Data sharing is not applicable to this article. Further inquiries can be directed to the corresponding authors.
